# An Overview of The Available Methods for Morphological Scoring of Pre-Implantation Embryos in *In Vitro* Fertilization

**DOI:** 10.22074/cellj.2015.486

**Published:** 2015-01-13

**Authors:** Nahid Nasiri, Poopak Eftekhari-Yazdi

**Affiliations:** Department of Embryology at Reproductive Biomedicine Research Center, Royan Institute for Reproductive Biomedicine, ACECR, Tehran, Iran

**Keywords:** Embryonic Development, Cleavage Stage, *In Vitro* Fertilization, Zygote, Blastocyst

## Abstract

Assessment of embryo quality in order to choose the embryos that most likely result in pregnancy is the critical goal in assisted reproductive technologies (ART). The current trend in human *in vitro* fertilization/embryo transfer (IVF/ET) protocols is to decrease the rate of multiple pregnancies after multiple embryo transfer with maintaining the pregnancy rate at admissible levels (according to laboratory standards). Assessment of morphological feathers as a reliable non-invasive method that provides valuable information in prediction of IVF/intra cytoplasmic sperm injection (ICSI) outcome has been frequently proposed in recent years. This article describes the current status of morphological embryo evaluation at different pre-implantation stages.

## Introduction

Accurate selection of embryos for transfer and prediction of implantation is the most important topic in assisted reproduction (1). Generally, quality and the rate of development in human embryos that are produced *in vitro* may vary widely. These differences may indicate the inherent diversity in the potential of gametes as well as in details of the *in vitro* fertilization (IVF) method and culture medium status (2). The success rate of IVF is mainly related to the number of embryos transferred as well as factors such as embryo quality, patient’s condition, and laboratory standards. A lower number of embryos can decrease the chance of pregnancy. However, if the goal is to increase pregnancy rate with restricting the possibility of multiple pregnancies, more sensitive and non invasive methods are required for embryo selection prior to transfer (3).

Application of a proper embryo scoring system has many potential benefits such as; 1. accurate selection of embryos prior to transfer, 2. reduction of the risk of multiple pregnancies, 3. assessment of different culture media and 4. comparison of embryo quality between patient cycles (4). It is clear that the use of such efficient methods are required for selection of proper embryo characteristics which are based on a foundation of basic research and credited by clinical studies (5, 6). Therefore, identification of these features and the methods of their assessment is one of the requisites for the IVF/ intra cytoplasmic sperm injection (ICSI) success rate, admission of single embryo transfer (SET) and a reduction in the risk of multiple pregnancies (7).

Current embryo grading systems differ with regards to selection of embryo stage and criteria for assessment of embryo quality. There are several stages for the evaluation of preimplantation embryo’s quality. In this study we have reviewed some main protocols (including important embryo traits and different scoring methods) in each stage.

## Discussion

According to specific standards and laboratory facilitates, embryologists apply different protocols. Each includes the proper embryo criteria and appropriate time point for quality evaluation of an embryo in their laboratory. However, all protocols fall into one of one of the following three stages.

### Quality assessment of zygote (16-18 hours after oocyte insemination)

Zygotes are formed after fusion of male and female gametes. In most assisted reproductive technology (ART) laboratories, the quality of male and female gametes (sperm, oocyte) is evaluated separately. For example, the abnormalities of oocyte morphology which are most frequently observed are large perivitelline space, dark zona pellucida, dark incorporations, spots, vacuoles, refractile bodies (dense and insoluble bodies which are produced within the cells) and irregular shape (8). Abnormal morphological criteria which can be observed in sperm consist of amorphous, round, large, small, vaculated or tapered head, neck and midpiece defects, excess residual cytoplasm and coiled, broken multi and short tail (9).

The first step for assessment of embryo quality is evaluation of zygotes or pronuclear stage embryo quality. In the recent years, there has been growing interest in the evaluation of pronuclear morphology to select the most competent embryos. For this purpose, *in vitro* fertilized human zygotes are classified on the basis of different features such as; number, equality, size and distribution of nucleoli, pronuclear size and alignment, the time of pronuclear breakdown and presence or absence of cytoplasmic halo (10-13).

Two main systems for evaluation of pronuclear stage morphology have been reported by Scott and Smith (10) and Tesarik et al. (14). In busy IVF laboratories, these systems are usually impossible to implement (with the exception of time-lapse technology which will be discussed later) because it has a very detailed classification and is time consuming.

**Fig 1:**
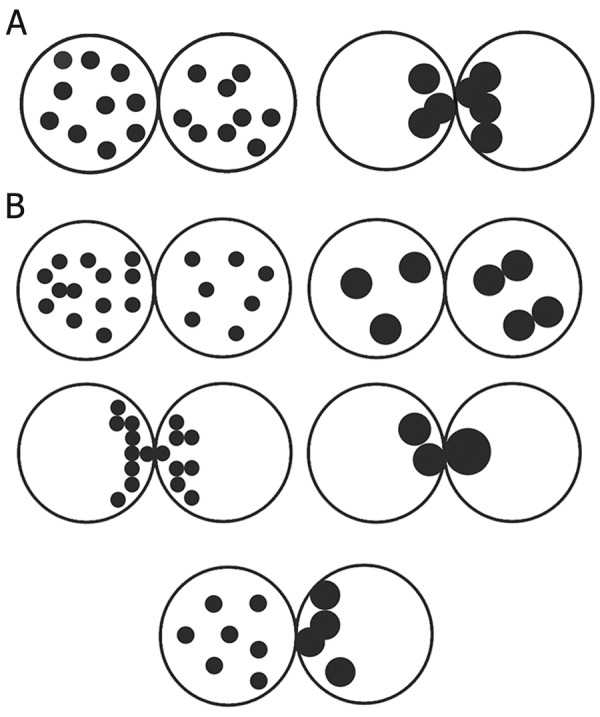
A. Classification of pronuclear morphology according to Brezinova et al. (18). Pattern " O", is defined as the same number of small nucleolar precursor bodies (NPBs) distributed in the nucleus or large NPBs with polar distribution between the two pronuclei. B. Classification of pronuclear morphology according to Brezinova et al. (18). Pattern " Other", Zygotes with nonsymmetrical alignments of NPB.

**Fig 2:**
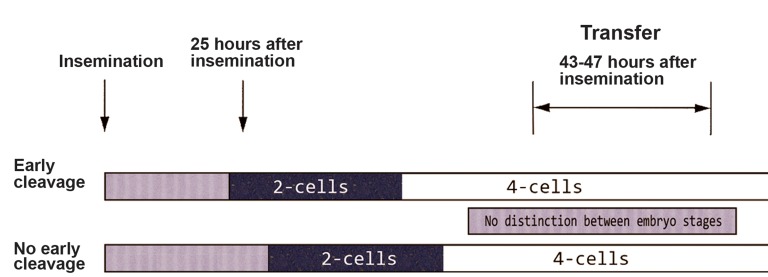
Embryo scoring based on specific time points for embryo cleavage during screening. Image obtained from the article of Brazinova et al. (18).

Tesarik and Greco (15) classified zygotes based on size and number as well as distribution of nucleoli or their precursors [nucleolar precursor bodies (NPBs)]. After this report, other simplified grading systems have been provided using the number, alignment and position of NPBs (16, 17). One example for such scoring system is the method reported by Brezinova et al. (18) in 2009. They classified zygotes into two different patterns ("O", "Other") based on pronuclei morphology of the zygote and an early cleavage rate. Pattern "O" consisted of zygotes that exhibited the same number of small NPBs distributed in the nucleus or large NPBs with polar distribution between the two pronuclei. Zygotes with non-symmetrical alignments of NPB achieved the "Other" score (Fig 1A, B). The second criterion in this assessment is the first mitotic division. This occurrence is checked 23-27 hours after insemination. At this moment, embryos with two blastomeres are classified as early cleavage (EC) embryos and those that do not reach this stage with intact nuclear membranes are classified as no early cleavage (NEC) embryos. The results indicate that best outcome can be achieved if both embryo scoring systems are used together and embryos are classified as EC and "O" pattern (18). EC embryos show more than two times the pregnancy rate and three times the implantation rate compared with non EC (NEC) embryos. These results have previously been proposed by Shoukir et al. (3) in 1997. They reported that fertilized embryos which cleaved to the 2-cell stage 25 hours after insemination were classified as EC embryos versus those that did not reach the 2 cell stage (NEC). In this study, EC embryos showed better pregnancy outcomes compared with NEC embryos. They proposed the EC definition method as a simple, effective noninvasive method for selection and assessment of embryos before transfer (Fig 2).

Pronuclear zygote morphology criteria according to a study by Depa-Martynow et al. (1) in 2007 included the presence of a cytoplasmic halo, nuclear size and alignment, NPB number and distribution. It has been said that in many mitotic cells, an equal number of NPB between the nuclei is a necessary event whereas an unequal number of NPB results in an abnormal cell cycle (19).

**Fig 3:**
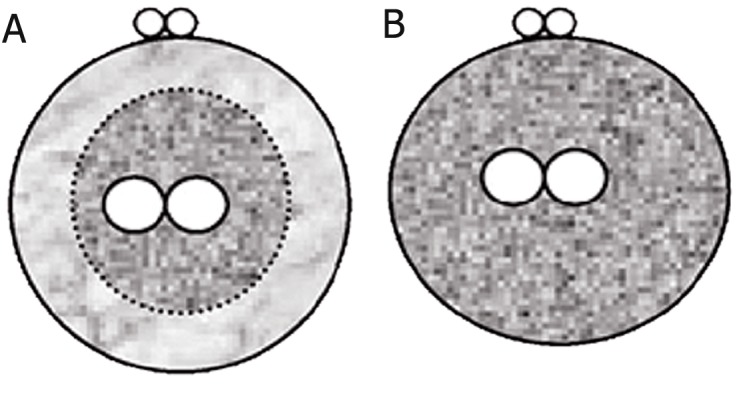
The presence or absence of a cytoplasmic halo: zygote with cytoplasmic halo (A); zygote without cytoplasmic halo (B). Classification according to Depa-Martynow et al. (1).

Cytoplasmic halo is another important criterion for pronuclear stage embryo grading and initially reported by Payne et al. (20) in 1997 as a sub plasmalemmal zone of a translucent cytoplasm immediately prior to formation of the male and female pronucleus. This structure often progresses to coat the entire cyto-cortex and is thought to be the result of a microtubuleorganized shift of the mitochondria and other cytoplasmic component to the center of the oocyte, so that no detectable mitochondria are found in the cortical region of the fertilized oocyte (21). It is possible that distribution of mitochondria to the perinuclear regions is involved in cell cycle regulation by Ca^2+^ cooperation and ATP release (22, 23). Location of immature mitochondria next to the pronuclei can lead to complete maturity of the mitochondria (23, 24). Figure 3 illustrates the pronuclear embryo classification based on the presence or absence of a cytoplasmic halo.

**Fig 4:**
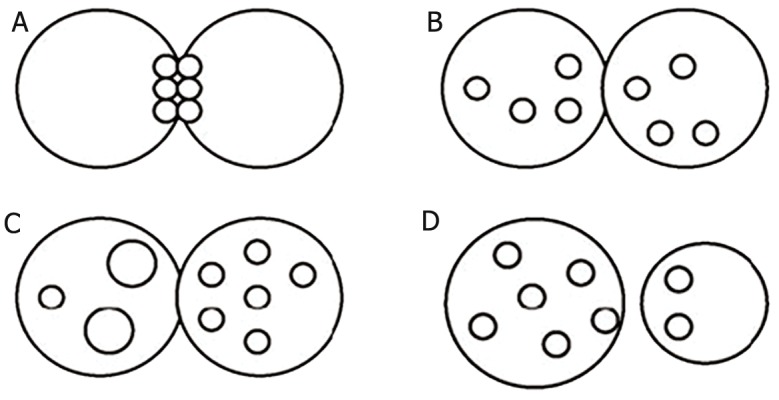
Zygote classification according to Scott et al. (19): Z_1_ zygote (equal numbers of nucleolar precursor bodies (NPBs) aligned at the pronuclear junction) (A), Z_2_ zygote (equal number and size of nucleoli which were scattered equally in the two nuclei) (B), Z_3_ zygote (zygotes with unequal numbers or size of nucleoli in just one nucleus and equal number and size of nucleoli in another nucleus) (C) and Z_4_ zygote (the pronuclei are located in the periphery or are separated with different sizes) (D).

Figure 4 shows the classification manner of zygotes according to the Z-scoring system proposed by Scott (19). This method is based on nuclear size and alignment as well as NPB number and distribution. In summary, Z_1_ zygotes have equal numbers of NPB aligned at the pronuclear junction (Fig 4A). Z_2_ zygotes have equal numbers and size of nucleoli (between 3 and 6) which are scattered equally in the two nuclei (Fig 4B). Z_3_ zygotes have equal numbers of NPB that are equal size in the same nucleoli but with one nucleus that is situated at the pronuclear junction and the other with nucleoli dispersion, as well as zygotes with unequal numbers or size of nucleoli (Fig 4C). Zygotes with pronuclei which are located periphery or are separated with very different size are classified as Z_4_ (Fig 4D).

**Fig 5:**
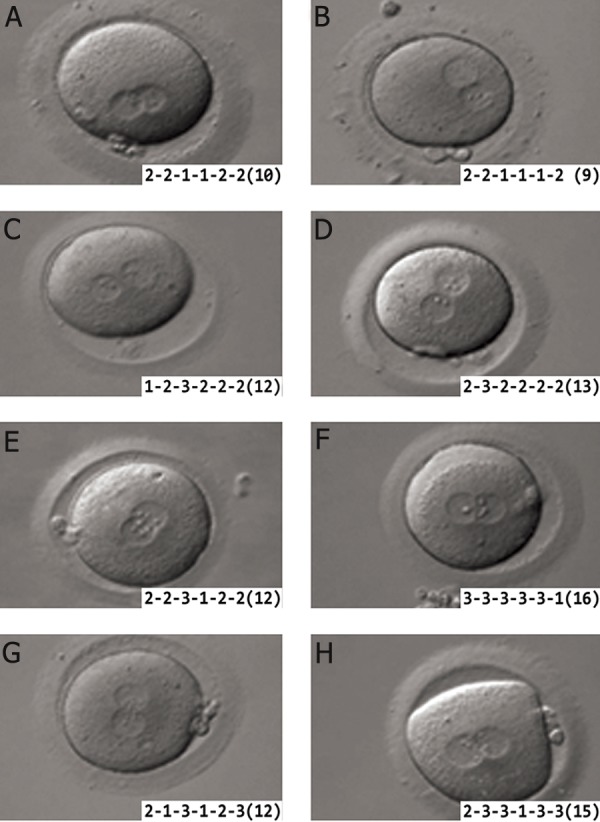
Examples of zygote scoring according to Senn et al. (25). Scores (1, 2 or 3) assigned to each individual parameter (proximity, orientation and centering of the pronuclei, cytoplasmic halo, number and polarization of nucleolar precursor bodies (NPBs)) are indicated for each zygote. The cumulated pronuclear score (CPNS) is indicated in parentheses. (Bar=10 μm).

Another method for qualitative classification of embryos at the 2PN stage has been proposed by Senn et al. (25) in 2005. In this method, zygotes are initially graded based on proximity, orientation and centering of the pronuclei, cytoplasmic halo, number and polarization of NPBs, then, the cumulated pronuclear score (CPNS) which is the sum of scores assigned to the six parameters is calculated for each zygote (Fig 5). It has been observed that lower CPNS values of frozen-thawed zygotes may indicate the freezing damage to zygotes, thus, CPNS may be used as a single predictor tool for implantation of both fresh and frozen-thawed zygotes (25). Figure 5 shows examples of zygote assigned scores (1, 2, 3) for zygotes and CPNS is indicated in parentheses. According to the results of Senn et al. (25) the patterns of NPBs and cytoplasmic halo appear as the most important predictive factor for implantation rate in both types (fresh and frozen-thawed) of zygotes.

### Morphological quality assessment of cleavage stage embryos (day 3 after insemination)

Quality assessment of cleavage stage embryos is a common method in embryo quality assessment accepted by numerous embryologists. For this aim, some morphological features have been suggested. The most notable of these features are: fragmentation rate (Fr), irregularities in blastomeres, multinucleation and the blastomere number.

**Fig 6:**
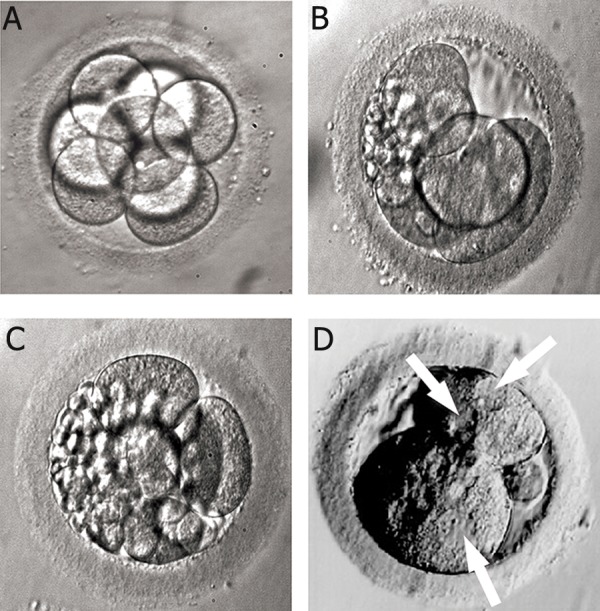
A. High quality 8-cell embryo. Embryo grading: 8 cell, grade 4. Grading method according to Advanced Fertility Center of Chicago (24). B. Irregular cells and fragmented 5-cell embryo. Embryo grading: 5 cell, grade 2. Grading method according to Advanced Fertility Center of Chicago (24). C. Severely fragmented and unevenly sized cells embryo. Embryo grading: 6 cell, grade 1. Grading method according to Advanced Fertility Center of Chicago (24). D. Multinucleated 2 cell embryo. Image obtained from the site of Advanced Fertility Center of Chicago (24).

Based on the "Advanced Fertility Center of Chicago" definition, several morphological criteria are considered in embryo classification; I. cell number: embryos should be 2 to 4 cells at 48 hours after egg retrieval and 7 to 10 cells by 72 hours (26) (Fig 6A). II. Cell regularity or degree of blastomere size equality (uneven blastomere cleavage): if individual cells are similar in size, the embryos have the best cell regularity. If they are approximately the same size, it is better to be compared with a different size (Fig 6B), III. degree of fragmentation: although the fragmentation phenomenon is totally common in human embryos, those with great than 25% fragmentation, have a low implantation potential (Fig 6C). IV. Presence of multinucleation: if there is more than one nucleus in each blastomere on either days 2 or 3, the embryo is multinucleated (Fig 6D) (26, 27). After day 3, it is highly difficult to identify multinucleation. Additional factors to be considered for grading and selection for transfers includes the presence of vacuoles, granularity and thickness of the zona pellucida, etc (28).

Generally, quality assessment of embryos is not performed until 48 hours after egg retrieval. In some IVF labs, zygotes on the first day after egg retrieval are assessed carefully. However 48 hours (day 2) embryos must have at least 2 cells and preferably 3 or 4 cells. For 72 hours (day 3) embryos, it is expected to observe of at least 6 cells and preferably 8 cells (26).

**Fig 7:**
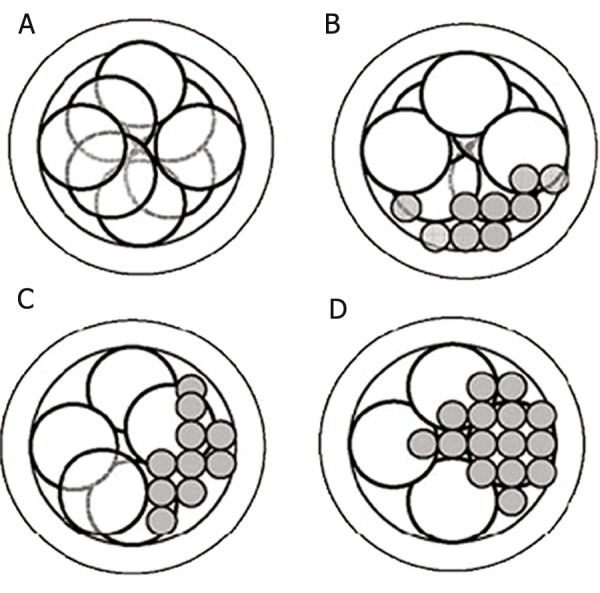
Embryos scoring according to Depa-Martynow et al. (1): grade A (embryos with 8 blastomeres and a maximum 20% of cytoplasmic fragmentation) (A), grade B (embryos with 8 blastomeres and over 20% cytoplasmic fragmentation) (B), grade C (4-6 cell embryos with a maximum 20% fragmentation) (C), grade D (4-6 cell embryos and over 20% fragmentation) (D).

Depa-Martynow et al. (1) in 2007 classified embryos based on morphological criteria either at the pronuclear stage or day 3 embryos (68 hours after insemination). Figure 7 shows their method for classification of day 3 embryos in four grades (A-D) according to the degree of cytoplasmic fragmentation and the number of blastomeres. The best embryos with at least 7 blastomeres (7-9 blastomeres) and maximum a 20% of cytoplasmic fragmentation are grade A. Grade B embryos have 7-9 cells with over 20% fragmentation. Grade C consists of 4-6 cells embryos with a maximum of 20% fragmentation. Grade D embryos are considered the worst quality with 4-6 cell embryos and over 20% fragmentation.

Another method for grading day 3 (65-75 hours) embryos has been developed by Desai et al. (4) in 2000. Their classification used unique features of this stage that consisted of cell number, fragmentation pattern, cytoplasmic pitting, compaction, equal sized blastomeres, blastomere expansion and absence of vacuoles. According to their results, although cell number and fragmentation pattern were good predictors of pregnancy outcome, the other day 3 specific parameters should be used for correct grouping of the embryos. Recent observations have suggested that the absolute amount of fragmentation (percentage of embryonic volume which is occupied by enucleate fragments) may contain less importance than the pattern of fragmentation (relative size and the spatial distribution of the fragments) (29). The loss of regulatory proteins during blastomere fragmentation may be one mechanism by which the developmental competence of an embryo is affected (30). Stensen et al. (31) in 2010 classified cleavage stage embryos based on the amount of fragmentation and blastomere size. Table 1 demonstrates their results.

**Table 1:** Morphological grading of embryos based on fragmentation and blastomere size according to Stensen et al. (31)


Score	Description

**3**	≥10-20% fragmentation, even or uneven blastomeres
**2**	>20-50% fragmentation, even or uneven blastomeres
**1**	Fragmentation precluded counting blastomeres
**0**	Cleavage arrest or morphologically abnormal embryo


In 2010 Pelinck et al. (32) have reported that, the cleavage rate plays an important role in quality assessment of a pre-implantation embryo before transfer. The embryo characteristic which is identified as the most optimal is the presence of 4 cells on day 2, 8 cells on day 3, less than 10% fragmentation and no multinucleated blastomeres (MNBs). In IVF-ET cycles, between embryos with the same age, those with more blastomeres are preferable for transfer. However slight fragmentation is a normal phenomenon in human embryos (29, 33, 34).

### Morphological quality assessment of blastocyst stage embryos (4- 5 days after fertilization)

When an embryo has developed to the stage of having 2 different cell components and fluid cavity, it becomes blastocyst (35). Usually, 4-5 days after fertilization, human embryos develop naturally to blastocyst stage in the body or from IVF in an IVF lab. Blastocyst transfer after IVF/ICSI can lead to a high pregnancy success rate with very low risk of multiple pregnancies; on the other hand, day 3 embryo morphology is insufficient for predict the implantation rate of the an embryo (35).

There are three distinguished parts in blastocyst structure for quality assessment, the two cell types, inner cell mass (ICM) and trophoectoderm (TE) and the fluid cavity. While the development of the blastocyst progresses, cells in the two regions divide and the fluid cavity enlarges. Many IVF clinics that transfer blastocysts use the blastocyst scoring system developed by Gardner et al. (36). This grading system has three separate quality scores for each blastocyst. I. Expansion and hatching manner II. ICM and III. TE (Tables 2, 3, 4).

**Table 2:** Embryo scoring based on blastocyst expansion grade according to Gardner et al. (36)


Expansion grade	Description

**1**	Blastocyst development and stage status
**2**	Blastocoel cavity more than half the volume of the embryo
**3**	Full blastocyst, cavity completely filling the embryo
**4**	Expanded blastocyst, cavity larger than the embryo, with thinning of the shell
**5**	Hatching out of the shell
**6**	Hatched out of the shell


**Table 3:** Blastocyst scoring based on inner cell mass (ICM) grade according to Gardner et al. (36)


ICM grade	ICM quality

**A**	Many cells, tightly packed
**B**	Several cells, loosely grouped
**C**	Very few cells


**Table 4:** BlastocystBlastocyst scoring based on trophoectoderm (TE) grade according to Gardner et al. (36)


TE grade	TE quality

**A**	Many cells, forming a cohesive layer
**B**	Few cells, forming a loose epithelium
**C**	Very few large cells


**Fig 8:**
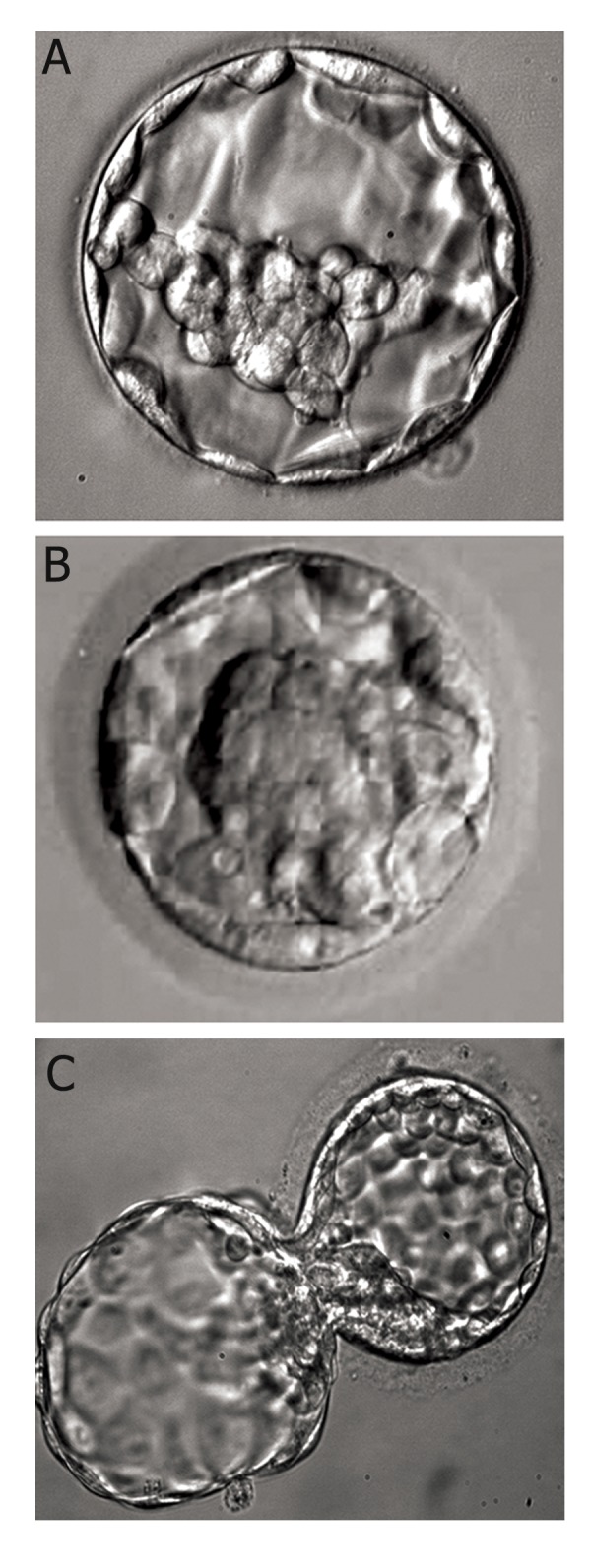
Examples of scored blastocyst according to Gardner et al. (36) and image obtained from our IVF laboratory (27). A. 4AB. 4: well expanded, A: ICM and B: TE. B. 1AB. 1: Cavity <1/2 of the embryo’s volume, A : ICM, B: TE. C. 5AA. 5: Blastocyst hatching out of shell, A: ICM, B: TE. ICM; Inner cell mass and TE; Trophoectoderm.

The final score assigned for each blastocyst is composed of these three scores. Therefore the first number is the expansion score, a number from 1-6 based on degree of expansion and the hatching status. The ICM score is listed second as, A. many cells forming a cohesive epithelium, B. few cells forming a loose epithelium and C. very few large cells. The final score is the TE score (A. tightly packed, many cells, B. loosely grouped, several cells and C. very few cells). For example, the triplex score of the blastocyst that is expanded, has many tightly packed cells in the ICM and a TE with a few cells and loose epithelium, is 4AB. Figure 8 are examples of blastocysts scored according to with this method.

#### Graduated embryo score (GES) and cumulative embryo score (CES)

**Fig 9:**
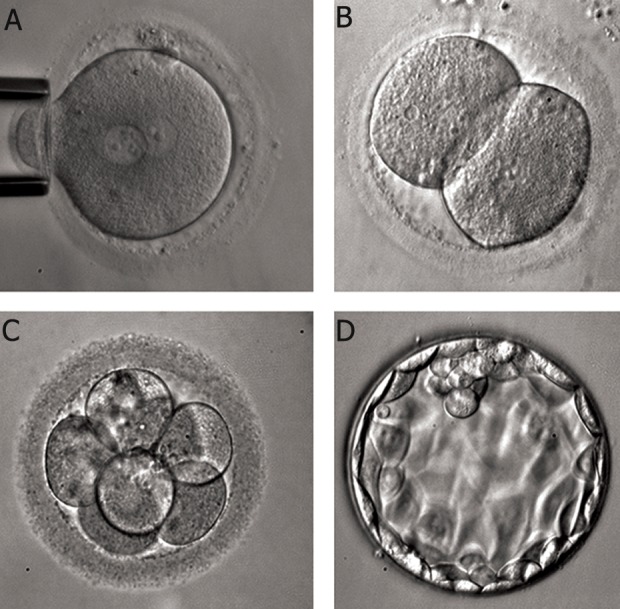
Embryo evaluation according to Fisch et al. (37). A. 16-18 hours post insemination (stage 1), nucleolar alignment along the pronuclear axi, B. 25-27 hours post insemination (stage 2), demonstrating symmetrical blastomere cleavage and no fragmentation, C. 64-67 hours post insemination (stage 3), demonstrating symmetrical cleavage, eight cells and no fragmentation and D. Expanded blastocyst at ~120 hours post insemination (stage 4) (Image obtained from our IVF laboratory).

Fisch et al. (37) in 2001 have proposed an embryo scoring system named the GES method. The GES system is composed of a group of assessments from the insemination at pronuclear stage morphology, then at early cleavage stage and finally on the 3^rd^ day after insemination. The GES system was introduced because in some embryos, limited features to the specific stage alone do not predict high implantation potential following IVF/ICSI. In the GES system, embryos are evaluated in four stages. Firstly , at 16-18 hours post-insemination, the cytoplasmic halo, vacuoles, pronuclear size, nucleolar alignment, polar body apposition and fragmentation are evaluated. The next evaluation occurs at 25-27 hours post insemination for dissolution of the blastomere cleavage, pronuclear membrane, degree and symmetry of fragmentation. The third evaluation performs 40-43 hours post-insemination and assesses the blastomere number, percentage and polarity of fragmentation. Blastomere number and morphology are evaluated 46-67 hours post insemination as the final step in this system. This method has been correlated with blastocyst development and implantation rate (38). In a study Fisch et al. (37) the researchers have emphasized some critical criteria in each stage after the results were analyzed such as; alignment of the nucleoli along the pronuclear axis at 16-18 hours post-insemination (Fig 9A), symmetrical cleavage and <20% fragmentation at the first zygote division (Fig 9B), and presence of 7-9 cells on day 3 (Fig 9C, D). However this sequential evaluation system requires more time, cost and manpower in an ART laboratory, in addition the frequent exclusion of embryos from an incubator is not negligible. The GES system was found to be a better predictor of pregnancy outcome than a single day assessment (37, 39).

The CES method is a mathematical scoring system proposed by Steer et al. (40) and is the sum of the scores of all embryos transferred. In this method, the score of each embryo on the day of transfer is obtained from multiplication of the morphological grade of the embryo by the number of blastomeres. The best outcome in terms of pregnancy rate is achieved when the CES is a maximum of 42. An increase in the amount of CES above 42 does not improve the pregnancy rate but enhances the rate of the multiple pregnancies. A method derived from the CES method, is the mean score of transferred embryos (MSTE) which is referred to as CES divided by the total number of transferred embryos as proposed by Terriou et al. (41) in 2001.

#### Time-lapse microscopy (TLM)

TLM is an ideal tool to record regular time interval photographs of an object such as a cell or an embryo over a period of several hours (42). This system is composed of four parts; a florescent/phase contrast microscope, a digital camera which records real-time images, computer software to control the camera and an incubator as an environment for preservation of the natural condition for cells or embryos.

Assessment of the oocyte/embryo developmental potential during fertilization, cleavage, development of the blastocyst, hatching and subsequent changes at intervals of 5-6 days, by the selection of credible morphological criteria and flexible evaluation using TLM instead of time point analysis may improve IVF success and reduce the risk of multiple pregnancies (7).

#### Other techniques for evaluation of embryo viability

In ART programs, the selection of an embryo with an acceptable implantation potential by means of methods that have high levels of clinical benefit and low level of potential risk for the embryo is of tremendous importance.

All methods which have been used for this goal are classified as either non-invasive or invasive. The embryo can be selected according to data derived from proteomic, genomic and/or metabolomic levels. An example of a non-invasive method is near infrared (NIR) spectroscopy of the embryos culture media which describes their metabolic profile as a viability index (43). Of note, some of these methods have been excluded due to the lack of achievement of desired results in a number of laboratories.

Invasive analysis of embryo viability can be performed by exclusion of one or two blastomeres of the 8-cell stage embryo or by the removal of TE cells in a blastocyst stage embryo (44). However removal of 2 cells from an 8 cell stage embryo is highly invasive and deleterious for the embryo. Any cell excluded by biopsy from a cleavage stage embryo may not be representative at the proteomic, genomic and transcriptomic levels because the information that can be derived from these cells is confused by the high incidence of mosaicism (45).

Selection of high quality embryos for transfer is currently based on morphological characteristics.

## Conclusion

We have carefully analyzed a number of primary scoring systems that are specific for different phases at the pre-implantation stage. Qualified assessment should rely on the combination of sequential pre-implantation embryo evaluation such that both zygote and pre-implantation steps should be evaluated for optimal and efficient selection of best embryo for transfer in IVF/ICSI cycles. On the other hand, the choice and evaluation of criteria most likely to increase the chance of implantation, is an important factor. For this purpose, assessment of specific time points for beginning embryo cleavage (EC or NEC embryos), the size and alignment of NPBs and presence or absence of a cytoplasmic halo are the most important properties for embryos in the zygote (PN) stage, whereas the blastomere size and equality, FR and multinucleation, are main features of cleavage stage embryos. Finally, blastocyst expansion and cell number are important criteria for blastocyst stage embryos.
